# Royal College of Psychiatrists (RCPsych) Dean's Grand Rounds – an Innovative Medical Education Tool to Bridge the Education-Practice Gap

**DOI:** 10.1192/bjo.2024.281

**Published:** 2024-08-01

**Authors:** Deepa Bagepalli Krishnan, Elizabeth Mullins, Subodh Dave

**Affiliations:** 1 Nottinghamshire Healthcare NHS Foundation Trust, Nottingham, United Kingdom; 2 University of Nottingham, Nottingham, United Kingdom; 3 Derbyshire Healthcare NHS Foundation Trust, Derby, United Kingdom

## Abstract

**Aims:**

RCPsych Dean's Grand Rounds focuses on understanding a problem or an opportunity for change in clinical practice using a patient story, academic evidence, and contextual data in this area to bridge the evidence-practice gap using a quality improvement approach. The Dean's Grand Rounds aims to embed the lived experience in clinical practice and use data to drive change.

**Methods:**

We held five virtual Dean's Grand Rounds with this format from June 2022 to January 2024. The sessions included a variety of medical professionals, carers and expert patients presenting on the chosen theme, followed by a panel discussion. The sessions were then made available for on-demand viewing via the RCPsych website for those unable to attend the live session. Qualitative and quantitative feedback helped us improve the sessions iteratively.

**Results:**

The sessions have enabled discussion of broader issues facing staff and patients, facilitating the exchange of ideas between professionals from divisions of the RCPsych from around the world. Participants globally attended these sessions, with over 1,000 registrations for the Grand Rounds on memory clinics and catatonia. The feedback for the sessions was overwhelmingly positive, with many participants praising the involvement of patients and carers and the opportunity to come together at the RCPsych level for learning. Many were attracted to the sessions because of the themes discussed, with 68.5% having excellent overall experience. Over 92% of the feedback participants thought the Grand Rounds had improved their professional practice. The majority of the feedback participants strongly agreed that lived experience is an important element in understanding the evidence-practice gap (4.4 on a Likert scale of 1, strongly disagree; 5, strongly agree) and that the Grand Rounds had enhanced their understanding of academic evidence and contextual data in the area (4.4, 4.39 respectively on a Likert scale of 1, strongly disagree; 5, strongly agree). The themes that stood out in the feedback were that participants liked the Grand Rounds format and were grateful to hear from patients, with suggestions to allocate more time for questions and answers. Learning from the feedback, we set up a resources page for each Grand Rounds to enable further learning.

**Conclusion:**

In their revitalised format, these sessions are proving highly effective in bringing the worldwide RCPsych community together to improve patient care and deliver educational and informative interactive content available on demand.

